# A *de novo* Variant of *ASXL1* Is Associated With an Atypical Phenotype of Bohring-Opitz Syndrome: Case Report and Literature Review

**DOI:** 10.3389/fped.2021.678615

**Published:** 2021-08-30

**Authors:** Weiqing Zhao, Xiao Hu, Ye Liu, Xike Wang, Yun Chen, Yangyang Wang, Hao Zhou

**Affiliations:** ^1^Department of Pediatrics, Guizhou Provincial People's Hospital, Guiyang, China; ^2^Department of Neurology, Guizhou Provincial People's Hospital, Guiyang, China; ^3^Department of Otolaryngology, Guizhou Provincial People's Hospital, Guiyang, China; ^4^Department of Gynecology, Guizhou Provincial People's Hospital, Guiyang, China

**Keywords:** Bohring-Opitz syndrome, *ASXL1* gene, intrauterine growth restriction, infant, rare diseases

## Abstract

Bohring-Opitz syndrome (BOS) is a rare genetic disease first reported by Bohring et al. in 1999. With the recent development of exome sequencing (ES), *de novo* truncating mutations in the additional sex-combs-like 1 (*ASXL1*) gene have been causally implicated in BOS. Herein, we describe a 7-month-old girl with intrauterine growth restriction, severe pulmonary infection, seizures, and craniofacial abnormalities (microcephaly, micro/retrognathia, hypertelorism, depressed nasal bridge, low-set ears and hypertrichosis) at birth. At a later stage, the patient developed global developmental delay. We performed ES and identified a *de novo* heterozygous mutation in *ASXL1*, namely, c.1210C>T/p.R404^*^. However, this case did not have trigonocephaly, facial hemangioma, prominent eyes, myopia, BOS posture, or brain abnormalities (enlarged subarachnoid spaces, agenesis of the corpus callosum, moderately enlarged cerebral ventricles, or prominent frontal subarachnoid spaces), which are common characteristics in most patients with BOS-harboring *ASXL1* mutations. These new data expand the phenotype of BOS driven by *ASXL1* and may assist in more accurately delineating the phenotypes caused by variants of this gene.

## Background

Bohring-Opitz syndrome (BOS) is a rare and severe autosomal dominant genetic disorder first reported by Bohring et al. (1). In 2011, Hastings et al. established clinical diagnostic criteria for this disease, including microcephaly, trigonocephaly, facial hemangioma, typical BOS posture, feeding difficulties, intrauterine growth restriction (IUGR), severe developmental delay, and characteristic craniofacial malformation (2). These common craniofacial features include prominent eyes, micro/retrognathia, and cleft palate. Some patients may have congenital anomalies (structural brain abnormalities, cardiac abnormalities, musculoskeletal abnormalities, etc.). Based on exome sequencing (ES), Hoischen and his colleagues reported an association between *ASXL1* mutation and BOS (3).

The human additional sex combs-like 1 (*ASXL1*) gene is located on chromosome 20q11, consists of 13 exons and 12 introns, and encodes a nucleoprotein with a length of 1,541 amino acids that is widely expressed in various tissues (4, 5). The N-terminus of the protein begins with the ASXN domain (also known as the HARE-HTH domain), which is predicted to promote interactions with DNA, and the ASXN domain is followed by the ASX homology (ASXH) domain (also known as the DEUBAD domain), which is encoded by exons 9–11 and participates in interactions with epigenetic regulatory proteins, including BRCA1-related protein 1 (BAP1). The plant homology domain (also known as the PHD domain), encoded by exon 13, is at the C-terminus and can bind methylated lysine. In general, the protein encoded by the *ASXL1* gene is involved mainly in epigenetic and transcriptional regulation. Notably, animal experiments have shown that the BOS phenotype occurs in *Asxl1*-knockout mice (6), and truncating variants in the *ASXL1* gene have been detected in cases that meet the clinical diagnostic criteria for BOS, suggesting a haploinsufficiency mechanism.

Cases of children with *ASXL1* variants have been reported primarily in Western countries, with only 7 cases reported in Asian countries [3 cases in China (7, 8), 1 in India (9), 1 in Japan (10), 1 in Turkey (11) and 1 in Korea (12)]. In this study, a Chinese child with BOS was found to carry an *ASXL1* gene variant. We conducted a systematic search and literature review to describe patients with BOS who had a clear genetic diagnosis, and we summarize the clinical manifestations and genetic variant information.

## Clinical Data

**Figure 1:**
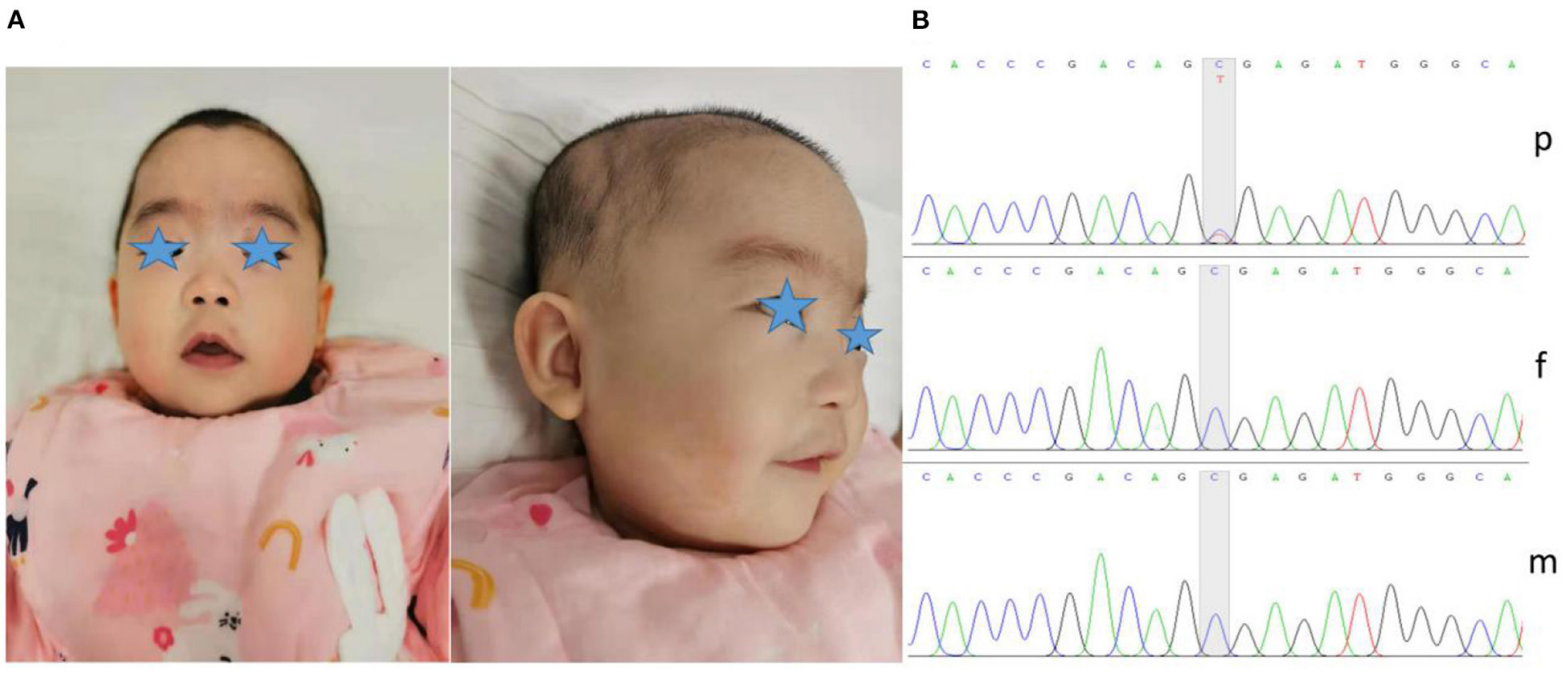
Dysmorphic features and variant analysis. **(A)** Photographs of our patient at 7 months showed microcephaly, micro/retrognathia, hypertelorism, depressed nasal bridge, low-set ears and hypertrichosis. **(B)** The proband carried the c.1210C>T variant in *ASXL1* (p), leading to a premature stop codon; however, her parents harbored no detectable variants (f and m).

The child in this case was a 7-month-old Chinese girl with a birth weight of only 2,230 grams (<-3 SD). During the neonatal period, the child underwent endotracheal intubation due to severe pneumonia and could not be removed from the ventilator for an extended period. The patient developed apnea, and caffeine citrate was used to excite the respiratory center. She had gastroesophageal reflux, so digestive tract malformations could not be excluded. In addition, the child experienced several seizures <10 days after birth; however, treatment with midazolam and phenobarbitone prevented further seizures, and she remained seizure free after treatment was stopped. The child developed anemia on the 12th day after birth, and a blood test showed a hemoglobin level of only 85 g/L. She was treated with a blood transfusion. At 7 months, when she returned to our department, the weight of the child was 5,000 grams (<-3 SD), her length was 60 cm (<-3 SD), and her head circumference was 38.4 cm (<-3 SD). She had microcephaly, hypertrichosis, gothic arch, depressed nasal bridge, hypertelorism, low-set ears, deep palmar creases, bent elbows, contractures of the toes, and hypotonia (Figure 1A). Her motor development was severely delayed, and she was unable to raise her head or sit on her own. The child subsequently underwent regular rehabilitation therapy. At the last follow-up, her language and motor function were still significantly delayed at the age of 1 year and 4 months, without any simple voiced sounds.

Her cerebrospinal fluid and cardiac ultrasound were normal during the neonatal period. At 6 months after birth, the total raw score for her fine motor ability test was 2, with a total percentage of 2.67% and ability value of 5.04; the total score for her Gross Motor Function Measure-88 test was 6.00, with a total percentage of 3.00% and ability value of 14.79; her Gesell developmental assessment result for personal social interaction was <4 weeks, with an adaptability of <4 weeks, a gross exercise equivalent to <4 weeks, a fine exercise equivalent to 8 weeks (development quotient 29.3), and a language ability area of <4 weeks. Both her electroencephalogram (EEG) and head magnetic resonance imaging (MRI) showed no abnormalities.

## Genetic Testing

To further clarify her diagnosis, after obtaining informed consent from the proband's guardian, exome sequencing was performed on DNA from the patient and her unaffected parents. Specifically, we collected 2 ml of peripheral blood from the patient and parents to extract DNA for ES. We screened for possible pathogenic variants and further verified the variants by Sanger sequencing. We finally identified one *de novo* heterozygous variant in the *ASXL1* gene, namely, c.1210C>T/p.R404^*^ (NM_015338.6) (Figure 1B). The variant was reported in three patients with BOS (3, 7, 13). According to the ACMG Classification Standards and Guidelines for Genetic Variations (14), the variant showed very strong evidence of pathogenicity since it was a null variant (PVS1), was reported in patients with BOS (PS1), and was a *de novo* variant (PS2). In conclusion, we regarded the variant identified in our patient as a pathogenic variant (PVS1+PS1+PS2).

## Literature Review

We searched the PubMed database, Human Gene Variant Database (HGMD), Online Mendelian Inheritance in Man (OMIM), and China National Knowledge Infrastructure (CNKI) using “Bohring-Opitz syndrome” and “*ASXL1*” as keywords. The search time was from the establishment of the database to June 30, 2021. Twenty documents were retrieved (3, 7–13, 15–26), including 1 Chinese document and 19 English documents. A total of 40 patients with BOS carried *ASXL1* gene variants, and their clinical characteristics are summarized in Table 1. Additionally, we used “*ASXL1*” to search for variants in the gene associated with BOS in the ClinVar database.

**Table 1:** Prevalence of clinical characteristics associated with *ASXL1* variants in patients diagnosed with BOS.

**Category**	**Total (%)**	**Present case**
**Sex**
Female	26/40 (65)	+
Male	12/40 (30)	
Unknown	2/40 (5)	
**Variant type**
Nonsense variant	23/40 (58)	+
Frameshift variant	17/40 (43)	
**Neonatal**
Respiratory distress	15/37 (41)	+
Feeding difficulties	32/34 (94)	+
IUGR	25/40 (63)	+
Infections	12/35 (34)	+
Apneas	8/32 (25)	+
**Craniofacial features**
Microcephaly	22/37 (59)	+
Trigonocephaly	18/37 (49)	-
Micro/retrognathia	25/36 (69)	+
Cleft palate	7/36 (19)	-
Gothic arch	22/28 (79)	+
Facial hemangioma	26/37 (70)	-
Hypertelorism	22/36 (61)	+
Prominent eyes	24/35 (69)	-
Upslanting palpebral fissures	14/36 (39)	-
Depressed nasal bridge	21/37 (57)	+
Anteverted nares	13/35 (37)	-
Low-set ears	22/37 (59)	+
Low hairline	17/30 (57)	+
Hypertrichosis	28/36 (78)	+
**Limbs and muscles**
BOS posture	24/37 (65)	-
Fixed contractures	23/38 (61)	+
Hypotonia	28/38 (74)	+
Syndactyly	6/37 (16)	-
Deep palmar creases	7/35 (20)	+
**Other**
Growth retardation	34/34 (100)	+
Strabismus	9/30 (30)	-
Myopia	18/30 (60)	-
Seizures	22/37 (59)	+
Genital abnormalities	6/37 (16)	-
Renal abnormalities	5/35 (14)	-
Cardiac abnormalities	14/37 (38)	-
Brain abnormalities	29/37 (78)	-
Tumor	2/36 (6)	-
Death	4/35 (11)	-

*Some clinical phenotypes are not fully characterized because the patients died before all features could develop or because these phenotypes are not mentioned in the literature*.

Including our patient, 40 patients with BOS carrying *ASXL1* variants have been reported to date. Among the 40 patients with BOS, there were 26 females, 12 males, and 2 patients without sex information (16, 17), with a female-to-male ratio of 2.2:1, and a total of 31 unique *ASXL1* truncating variants were found. Among these variants, *de novo* patterns occurred in 38 patients; one patient inherited the variant from a mosaic mother (with an allele fraction of 36%), and the other variant remained of unknown origin. However, there were seven reported recurrent variants, including c.1210C>T/p.R404^*^ (4 cases), c.1934dupG/p.G646Wfs^*^12 (2 cases), c.2324T>G/p.L775^*^ (2 cases), c.2332C>T/p.Q778^*^ (2 cases), c.2535dup/p.S846Qfs^*^5 (2 cases), c.2893C>T/p.R965^*^ (2 cases) and c.4060G>T/p.E1354^*^ (2 cases). The most common clinical presentations of patients with BOS with *ASXL1* gene variants were growth impairment (34/34, 100%), feeding difficulties (32/34, 94%), gothic arch (22/28, 79%), hypertrichosis (28/36, 78%), brain abnormalities (29/37, 78%), hypotonia (28/38, 74%), facial hemangioma (26/37, 70%), micro/retrognathia (25/36, 69%), prominent eyes (24/35, 69%), BOS posture (24/37, 65%), IUGR (25/40 63%), fixed contractures (23/38, 61%), hypertelorism (22/36, 61%), myopia (18/30, 60%), microcephaly (22/37 59%) and seizures (22/37 59%). The reported variants are listed in the Table 2.

**Table 2:** Previously reported BOS cases and the present case carrying *ASXL1* variants.

**Patient**	**Age**	**Nucleotide change**	**Amino acid change**	**References**
1	7 y	c.1049G>A	p.W350^*^	(8)
2	7 m	***c.1210C>T***	***p.R404*** ^*^	Present case
3	2 m	***c.1210C>T***	***p.R404*** ^*^	(7)
4	6 y	***c.1210C>T***	***p.R404*** ^*^	(13)
5	7 y	***c.1210C>T***	***p.R404*** ^*^	(3)
6	Unknown	c.1269dupT	p.L424fs	(17)
7	1 y	c.1272_1273delGT	p.Y425Qfs^*^12	(21)
8	13 y	c.1517_1518delGA	p.R506Nfs^*^3	(26)
9	5 y	c.1720–2A>G	p.I574Vfs^*^22	(22)
10	5 y	c.1867C>T	p.Q623^*^	(26)
11	3 m	c.1924G>T	p.G642^*^	(21)
12	died at 60 days	***c.1934dupG***	p.G646Wfs^*^12	(10)
13	4 y	***c.1934insG***	p.G646Wfs^*^12	(11)
14	11 y	c.2013_2014del	p.C672Wfs^*^4	(21)
15	7 y	c.2033dupG	p.R678fs^*^6	(15)
16	died at 11 m	c.2036_2037insG	p.G680Rfs^*^38	(9)
17	5 y	c.2100dupT	p.P701Sfs^*^16	(23)
18	died at 23 h after birth	c.2197C>T	p.Q733^*^	(3)
19	3 y	***c.2324T>G***	***p.L775*** ^*^	(3)
20	12 y	***c.2324T>G***	***p.L775*** ^*^	(3)
21	5 y	***c.2332C>T***	***p.Q778*** ^*^	(26)
22	3.5 y	***c.2332C>T***	***p.Q778*** ^*^	(3)
23	3 y	c.2407_2411del5	p.Q803Tfs^*^17	(25)
24	14 y	c.2468T>G	p.L823^*^	(3)
25	7 m	***c.2535dup***	p.S846Qfs^*^5	(21)
26	24 y	***c.2535dup***	p.S846Qfs^*^5	(3)
27	3 y	c.2759_2762dup	p.V922Ifs^*^3	(21)
28	died at 6 y	c.2773C>T	p.Q925^*^	(3)
29	21 m	c.2689delC	p.H897Ilefs^*^11	(8)
30	3 y	***c.2893C>T***	p.R965^*^	(20)
31	7 y	***c.2893C>T***	p.R965^*^	(25)
32	3 y	c.3077del	p.G1026Dfs^*^21	(21)
33	2.5 y	c.3083C>A	p.S1028^*^	(3)
34	10 m	c.3115C>T	p.Q1039^*^	(12)
35	24 y	c.3856C>T	p.Q1286^*^	(16)
36	12 y	***c.4060G>T***	p.E1354^*^	(26)
37	7 y	***c***.4060***G>T***	p.E1354^*^	(18)
38	17 y	c.4116_4117delTT	p.F1373fs	(19)
39	18 y	c.4198G>T	p.E1400^*^	(24)
40	22 y	c.4243C>T	p.R1415^*^	(16)

*Recurrent variants are indicated with bold italics; y, year; m, month*.

* *, termination codon*.

In total, 44 BOS patients with *ASXL1* gene variants were retrieved from the ClinVar database. There were 42 unique *ASXL1* variants, and 2 variants were observed in two patients: c.1210C>T/p.R404^*^, c.1934dupG/p.G646Wfs^*^12. There were 10 gene variants overlapping with our literature search, and they were c.1210C>T/p.R404^*^, c.1934dupG/p.G646Wfs^*^12, c.2036dupG/p.G680Rfs^*^38, c.2197C>T/p.Q733^*^, c.2407_2411del5/p.Q803Tfs^*^17, c.2535dupC/p.S846Qfs^*^5, c.2773C>T/p.Q925^*^, c.2893C>T/p.R965^*^, c.3083C>A/p.S1028^*^, and c.4060G>T/p.Q1354^*^. More importantly, 19 variants that were rated as pathogenic/likely pathogenic have not been reviewed in the literature, as shown in Table 3.

**Table 3:** *ASXL1* gene variants in patients diagnosed with BOS syndrome in the ClinVar database.

**Accession**	**Nucleotide change**	**Amino acid change**	**Clinical significance (Last reviewed)**
VCV000338072	c.-88_-86GCC	p.(=)	Uncertain significance (2016)
VCV001031903	c.69delC	p.Y24Tfs^*^6	Likely pathogenic (2018)
VCV000488471	c.217A>T	p.K73^*^	Pathogenic (2017)
VCV000998008	c.643G>A	p.A215T	Likely pathogenic (2019)
VCV000998006	c.658C>T	p.Q220^*^	Likely pathogenic (2019)
VCV000288745	c.1162_1163delGT	p.V388Pfs^*^21	Pathogenic (2016)
VCV000030986	c.1210C>T	p.R404^*^	Pathogenic (2019)
VCV001030494	c.1225A>G	p.K409E	Uncertain significance (2020)
VCV000598750	c.1283_1284delAG	p.Q428Rfs^*^9	Pathogenic (2018)
VCV001031900	c.1387A>G	p.S463G	Uncertain significance (2018)
VCV000803604	c.1426_1427dupGT	p.E477Wfs^*^227	Pathogenic (2019)
VCV001120115	c.1567A>T	p.K523^*^	Likely pathogenic (2020)
VCV001031901	c.1589C>T	p.A530V	Uncertain significance (2018)
VCV001174493	c.1719+1G>A		Pathogenic (2021)
VCV000870588	c.1720-1G>A		Pathogenic (2019)
VCV000426927	c.1934dupG	p.G646Wfs^*^12	Pathogenic/Likely pathogenic (2018)
VCV000807543	c.2036dupG	p.G680Rfs^*^38	Pathogenic (2019)
VCV000030989	c.2197C>T	p.Q733^*^	Pathogenic (2011)
VCV000039468	c.2407_2411del5	p.Q803Tfs^*^17	Pathogenic (2012)
VCV000694695	c.2416_2417dupAC	p.V807Pfs^*^12	Pathogenic (2019)
VCV000030988	c.2535dupC	p.S846Qfs^*^5	Pathogenic (2011)
VCV000338100	c.2544A>T	p.T848=	Likely benign (2018)
VCV000030985	c.2773C>T	p.Q925^*^	Pathogenic (2011)
VCV000338102	c.2802T>C	p.A934=	Likely benign (2016)
VCV000039469	c.2893C>T	p.R965^*^	Pathogenic (2016)
VCV000030987	c.3083C>A	p.S1028^*^	Pathogenic (2011)
VCV000431709	c.3202C>T	p.R1068^*^	Pathogenic (2017)
VCV000338106	c.3212C>T	p.A1071V	Likely benign (2016)
VCV000338107	c.3351C>A	p.P1117=	Uncertain significance (2016)
VCV000982935	c.3425A>G	p.Q1142R	Uncertain significance (2019)
VCV001031902	c.3437C>A	p.S1146^*^	Pathogenic (2018)
VCV000803605	c.3460G>A	p.G1154S	Uncertain significance (2019)
VCV000632647	c.3637del	p.L1213fs	Likely Pathogenic (2018)
VCV000560957	c.3700C>T	p.Q1234^*^	Pathogenic (2017)
VCV000488446	c.3754_3758del	p.Q1251Pfs^*^10	Pathogenic (2017)
VCV000666295	c.3769delG	p.A1257Lfs^*^23	Likely Pathogenic (2015)
VCV001030495	c.3946C>G	p.R1316G	Uncertain significance (2019)
VCV000976125	c.4048C>T	p.Q1350^*^	Pathogenic (2017)
VCV000632646	c.4060G>T	p.E1354^*^	Pathogenic (2018)
VCV000548554	c.4109AGA	p.K1371del	Uncertain significance (2018)
VCV000587611	c.4282TCT	p.S1429del	Uncertain significance (2018)
VCV000338140	c.1556_1557delCC	p.P519Qfs^*^9	Uncertain significance (2016)

* *, termination codon; =, synonymous mutation*.

## Discussion

The current patient was a full-term small child who had IUGR, severe infection, difficulty feeding during the neonatal period, abnormal facial morphology, severe developmental delay, microcephaly and characteristic dysmorphic features. However, it is worth noting that this patient did not have trigonocephaly, facial hemangioma, prominent eyes, myopia, BOS posture (which is a typical posture of flexing the elbows and wrists and ulnar deviation of the wrists and metacarpophalangeal joints), or brain abnormalities, which are common characteristics in most patients with *ASXL1* pathogenic variants. Although our case did not meet the clinical diagnostic criteria for BOS established by Hastings et al. (2), the patient obtained molecular diagnosis through genetic testing showing a *de novo* heterozygous variant c.1210C>T/p.R404^*^ in the *ASXL1* gene.

BOS, which was originally reported as Oberklaid-Danks syndrome, is a rare genetic disease that was first distinguished from Opitz Trigonocephaly C syndrome by Dr. Axel Bohring in 1999. Bohring et al. reported four cases of premature cranial suture closure, orbital hypertelorism, prominent eyes, cleft lip and palate, limb abnormalities, feeding difficulties, and severe developmental delay. Based on the most common phenotype, the diagnosis of Bohring-Opitz syndrome (BOS) was later determined using established diagnostic criteria (2). To date, more than 50 patients with BOS meeting the clinical diagnostic criteria have been reported (19, 21). According to previous research, BOS is caused by *de novo* heterozygous mutations in the *ASXL1* gene; however, only 39 patients with BOS and a definite molecular diagnosis have been reported in the literature. Nonsense variants are the most common variant type, resulting in premature termination of protein synthesis and loss of *ASXL1* protein function, suggesting that *ASXL1* loss of function is most likely the disease-causing mechanism (27).

Thus, far, the c.1210C>T/p.R404^*^ variant seems to be the most common *ASXL1* gene variant in patients with BOS. The specific variant from the proband has now been reported in 4 cases in the literature (4/40, 13.8%), as well as additional, likely unreported cases in ClinVar. All 4 patients with this gene variant were female, and the most common clinical features with this gene variant included growth impairment (4/4, 100%), feeding difficulties (4/4, 100%), microcephaly (4/4, 100%), hypertrichosis (4/4, 100%), IUGR (3/4, 75%), infections (3/4, 75%), micro/retrognathia (3/4, 75%), gothic arch (3/4, 75%), seizures (3/4, 75%), and hypotonia (3/4, 75%). In contrast, trigonocephaly (1/4, 25%), hypertelorism (1/4, 25%), prominent eyes (1/4, 25%), and BOS posture (1/4, 25%) were less common in patients with this gene variant.

Two patients with BOS who carry *ASXL1* variants have been diagnosed with Wilms tumor, bringing the incidence of kidney tumors to 2/36 (6%) in *ASXL1* variant-positive patients with BOS. *ASXL1*, which has been mapped to chromosome 20q11.21, encodes the additional sex combs-like protein 1. ASXL1 belongs to the Trithorax (TrxG) and polycomb group (PcG) families (27), suggesting that this protein is required for maintenance of both activation and silencing of Hox genes (5, 28). Deletions of *ASXL1* have been described in myelodysplastic syndrome and other myeloid malignancies, suggesting that ASXL1 plays a role in transcriptional activation and repression (28, 29). Numerous studies have confirmed that *ASXL1* gene variants are detected in nearly all types of myeloid tumors and that the variant rate is above 5% (27). In addition, *ASXL1* variants play an important role in malignant tumors that occur in other systems of the human body (30). For example, high variant rates in various solid tumors, such as breast cancer and colon cancer, have been reported. Therefore, researchers have suggested that abdominal ultrasound should be performed in patients with BOS every 3–4 months during the first 8 years of life (21). Four patients with BOS died in early childhood, and two of them died from bradycardia, obstructive apnea, or lung infections (3, 9, 10); however, there was no definite correlation between the incidence of Wilms tumor and mortality.

In conclusion, in view of phenotypic heterogeneity, the clinical management of BOS is highly challenging. Here, we have reviewed the most common clinical features of patients with BOS who carry *ASXL1* variants, which may help improve the understanding of the phenotype-genotype correlation. However, the correlations need to be studied with a larger patient cohort in the future.

## Data Availability Statement

The datasets presented in this study can be found in online repositories. The names of the repository/repositories and accession number(s) can be found in the article/supplementary material.

## Ethics Statement

The studies involving human participants were reviewed and approved by Guizhou Provincial People's Hospital. Written informed consent to participate in this study was provided by the participants' legal guardian/next of kin. Written informed consent was obtained from the individual(s), and minor(s)' legal guardian/next of kin, for the publication of any potentially identifiable images or data included in this article.

## Author Contributions

WZ and XH drafted the manuscript. WZ, XH, YL, XW, YC, and YW contributed to the clinical data acquisition. YC and HZ contributed to the analysis and genetic evaluation. HZ critically revised the manuscript. All authors contributed to the article and approved the submitted version.

## Conflict of Interest

The authors declare that the research was conducted in the absence of any commercial or financial relationships that could be construed as a potential conflict of interest.

## Publisher's Note

All claims expressed in this article are solely those of the authors and do not necessarily represent those of their affiliated organizations, or those of the publisher, the editors and the reviewers. Any product that may be evaluated in this article, or claim that may be made by its manufacturer, is not guaranteed or endorsed by the publisher.
